# Serum Fibroblast Growth Factor 21 Is Markedly Decreased following Exercise Training in Patients with Biopsy-Proven Nonalcoholic Steatohepatitis

**DOI:** 10.3390/nu15061481

**Published:** 2023-03-20

**Authors:** Jonathan G. Stine, Jaclyn E. Welles, Shelley Keating, Zeba Hussaini, Christopher Soriano, J. Wes Heinle, Nathaniel Geyer, Vernon M. Chinchilli, Rohit Loomba, Scot R. Kimball

**Affiliations:** 1 Division of Gastroenterology and Hepatology, Department of Medicine, Penn State Health Milton S. Hershey Medical Center, Hershey, PA 17033, USA; 2 Department of Public Health Sciences, College of Medicine, The Pennsylvania State University, Hershey, PA 17033, USA; 3 Liver Center, Penn State Health Milton S. Hershey Medical Center, Hershey, PA 17033, USA; 4 Cancer Institute, Penn State Health Milton S. Hershey Medical Center, Hershey, PA 17033, USA; 5 Department of Cellular and Molecular Physiology, College of Medicine, The Pennsylvania State University, Hershey, PA 17033, USA; 6 School of Human Movement and Nutrition Sciences, The University of Queensland, St Lucia, QLD 4072, Australia; 7 Division of Gastroenterology and Hepatology, Department of Medicine, University of California San Diego, San Diego, CA 92093, USA; 8 NAFLD Research Center, University of California San Diego, San Diego, CA 92093, USA

**Keywords:** nonalcoholic fatty liver disease, fatty liver, physical activity, cardiorespiratory fitness, biomarker

## Abstract

Background and Aims: Exercise remains a key component of nonalcoholic fatty liver disease (NAFLD) treatment. However, mechanisms underpinning the improvements in NAFLD seen with exercise are unclear. Exercise improved liver fat and serum biomarkers of liver fibrosis in the NASHFit trial. We investigated exercise’s mechanism of benefit by conducting a post hoc analysis of these data to determine the relationship between serum fibroblast growth factor (FGF) 21, which is implicated in NAFLD development, and exercise. Methods: In the 20 wk NASHFit trial, patients with nonalcoholic steatohepatitis (NASH) were randomized to receive moderate-intensity aerobic exercise training or standard clinical care. Mediterranean-informed dietary counseling was provided to each group. Change in serum FGF21 was measured after an overnight fast. Results: There was a significant improvement in serum FGF21 with exercise training compared to standard clinical care (*p* = 0.037) with serum FGF21 reducing by 22% (−243.4 +/−349 ng/mL) with exercise vs. a 34% increase (+88.4 ng/mL +/−350.3 ng/mL) with standard clinical care. There was a large inverse association between change in serum FGF21 and change in cardiorespiratory fitness (VO_2_peak) (r = −0.62, 95% CI −0.88 to −0.05, *p* = 0.031), and on multivariable analysis, change in VO_2_peak remained independently associated with change in FGF21 (β = −44.5, 95% CI −83.8 to −5.11, *p* = 0.031). Conclusions: Serum FGF21 is markedly decreased in response to aerobic exercise training, offering a novel mechanism to explain the observed reduction in liver fat and improvement in serum biomarkers of liver fibrosis in patients with NASH who do exercise.

## 1. Introduction

Globally, nonalcoholic fatty liver disease (NAFLD) is a leading cause of chronic liver disease and affects between 25% and 30% of the world population [1]. Beyond the vast prevalence rates, NAFLD is also independently associated with lower overall survival [2]. While multiple factors contribute to NAFLD development, physical inactivity plays a major role and leads to abnormal accumulation of fat in the liver [3,4]. NAFLD incorporates two distinct histologic processes including NAFL, the non-progressive type, and nonalcoholic steatohepatitis (NASH) [5]. If uncorrected, NASH can lead to the development of liver fibrosis and disease progression to cirrhosis or hepatocellular carcinoma, often necessitating liver transplantation [6,7].

In the absence of a regulatory-agency-approved drug therapy, lifestyle modification through dietary change and increased physical activity remain the most effective treatment for NAFLD and NASH [8]. Physical activity, including exercise training which is a type of physical activity that is planned, structured, and repetitive [9], has many beneficial effects on the liver, including a reduction in liver fat, and when coupled with modest weight loss may halt or reverse liver fibrosis [8,10,11,12]. Physical activity also has many extrahepatic benefits, including improvement in cardiorespiratory fitness, favorable change in body composition with loss of body fat and gain of skeletal muscle, improvement in hemostasis, gain in bone density, and reduction in metabolic risk [8,11,12]. However, despite our longstanding knowledge about the extensive clinical benefits of physical activity and exercise training, the mechanism explaining exercise’s benefit remains unknown.

Fibroblast growth factor (FGF) 21 is a hepatokine that regulates carbohydrate, lipid, and energy metabolism [13]. FGF21 is implicated in NAFLD development and disease progression to NASH; in fact, NAFLD and NASH are felt to be FGF21-resistant states, where an elevated serum FGF21 is observed as a compensatory response [14,15]. FGF21 has been identified as a therapeutic target, and early-phase trials in patients with NASH are underway or have been completed [16,17,18]. Importantly, FGF21 is also impacted by physical activity, and this intervention may reverse the FGF21-resistant state characteristic of NAFLD. While acute exercise may increase serum FGF21 owing largely to skeletal muscle stimulated production, over time, exercise training leads to physiologic adaptation, and a different impact on serum FGF21 is seen [19]. In fact, animal models of NAFLD have demonstrated that exercise training can significantly decrease serum FGF21 [20]. The improvement in FGF21 with aerobic exercise was confirmed in a recent study in elderly Japanese men without established NAFLD who underwent a short-term, five-week moderate-to-vigorous intensity aerobic exercise training program [21]. Whether this improvement is sustained with long-term aerobic exercise training in patients with established NAFLD/NASH is unknown, as the only study performed to date in patients with NAFLD that demonstrated a reduction in serum FGF21 utilized a 12-week resistance training program and not aerobic exercise [22].

Exercise training may also impact the FGF21–adiponectin axis, where an elevation in this ratio is observed in patients with NAFLD and indicative of metabolic dysfunction. In fact, FGF21/adiponectin ratio has been proposed as a NAFLD biomarker [23]. While animal models suggest that exercise training can protect against FGF21–adiponectin axis impairment, this remains largely unexplored in human subjects [24].

In the NASHFit trial, aerobic exercise training was shown to improve liver fat, serum biomarkers of liver fibrosis, as well as cardiorespiratory fitness [12], and to further investigate this significant, unanswered question, we conducted a post hoc analysis of these data to determine the relationship between serum FGF21 and long-term aerobic exercise training in patients with NASH. We also investigated the impact of aerobic exercise training on the FGF21–adiponectin axis.

## 2. Patients and Methods

### 2.1. Study Design and Population

This is a post hoc analysis of the NASHFit trial (NCT03518294) for which data on primary and secondary outcome measures were previously published [12]. The NASHFit trial compared the efficacy of 20 weeks of moderate-intensity aerobic exercise training to standard clinical care. Of the 28 patients enrolled, 24 patients completed the trial between May 2018 and February 2021. All patients provided informed consent prior to being included in the study. The study was approved by the Penn State Health Institutional Review Board (Study 8507). All research methods were in accordance with the Declaration of Helsinki, Good Clinical Practice guidelines, and local regulatory requirements. Specific details about subject recruitment, randomization, sample size, and other methods were provided in previous papers [12,25]. Inclusion criteria included sedentary adults with biopsy-confirmed NASH using the NASH Clinical Research Network histological scoring system [26]. Patients were excluded for uncontrolled diabetes, other chronic liver disease, excessive alcohol consumption, or an inability to perform regular exercise. Full eligibility criteria were previously published [12,25]. Patients were randomized 2:1 to intervention with exercise training or a standard of care group using a list generated by computer randomization (REDCap, Vanderbilt University) [27]. No stratified randomization was performed. Patients in the intervention group performed five moderate-intensity aerobic exercise sessions (45–55% VO_2_peak) per week, each lasting 30 min. Standard of care control subjects were instructed to continue their current clinical care. Compliance was ensured by remote monitoring with fitness activity trackers and direct supervision of exercise training sessions. Both study groups received Mediterranean-based dietary counseling. Despite this counseling, no change in dietary practices was observed in the NASHFit trial, with similar macronutrient intake reported before and after intervention, and no clinically significant changes in body weight were observed for either group [12].

### 2.2. Laboratory Methods

Blood samples from the NASHFit trial were collected after an overnight fast and, after processing, immediately stored at −80 °C. FGF21 levels were assessed using a Human FGF21 Quantikine enzyme-linked immunoassay (ELISA) kit (catalog number DF2100) from R&D Systems (Minneapolis, MN, USA) following the manufacturer’s instruction.

### 2.3. Statistical Analysis

For this post hoc study, the main outcome of interest was change in serum FGF21. This was performed with the use of paired and two-sample *t*-tests. Both between-group and within-group comparisons were performed where appropriate. Statistical significance was defined by two-sided *p*-values of <0.05. Secondary outcomes are presented as geometric means with 95% confidence intervals (CIs) or median with interquartile ranges, if negative or zero values. Both between- and within-group comparisons were performed. Continuous endpoints were analyzed with the use of paired and two-sample *t*-tests and categorical endpoints by the chi-squared test and Fisher’s exact test, where appropriate. Pearson’s correlation coefficients were calculated between FGF21 and routinely captured clinical variables from the NASHFit study. Linear regression modeling was performed to determine predictors of FGF21 change. Variables included in the final model were change in visceral adipose tissue, magnetic resonance imaging proton density fat fraction (MRI-PDFF), and maximal oxygen uptake (VO_2_peak). SAS (Cary, NC, USA) Version 9.4 was used for all statistical analysis.

## 3. Results

### 3.1. Baseline Characteristics

Of the 24 patients who completed the NASHFit trial, 20 were included in this analysis (12 exercise, 8 standard of care controls) where serum FGF21 could be measured. The four patients without serum FGF21 were similar in baseline characteristics to the included patients. For patients with measurable serum FGF21, mean patient age was 52 +/−12 years (range 25 to 69 years). Mean body weight was 100.1 +/−18.5 kg, and mean body mass index (BMI) was 32.8 +/− 5.2 kg/m^2^. The majority of patients were female (55%). In terms of metabolic comorbidities, 80% had hypertension, 60% had hyperlipidemia, and 45% had diabetes. Liver fibrosis stage was as follows: 55% (*n* = 11) F0/F1 fibrosis, 25% stage F2 (*n* = 5), 15% stage F3 (*n* = 3), and 5% stage F4 (*n* = 1). Demographic and baseline clinical characteristics were similar between the exercise and the standard of care control group (Table 1). Importantly, the two groups were well matched for age, sex, BMI, metabolic risk, and NASH phenotyping, to include both serum and imaging biomarkers as well as liver histology.

### 3.2. Change in Serum FGF21 following Exercise Training

**Figure 1:**
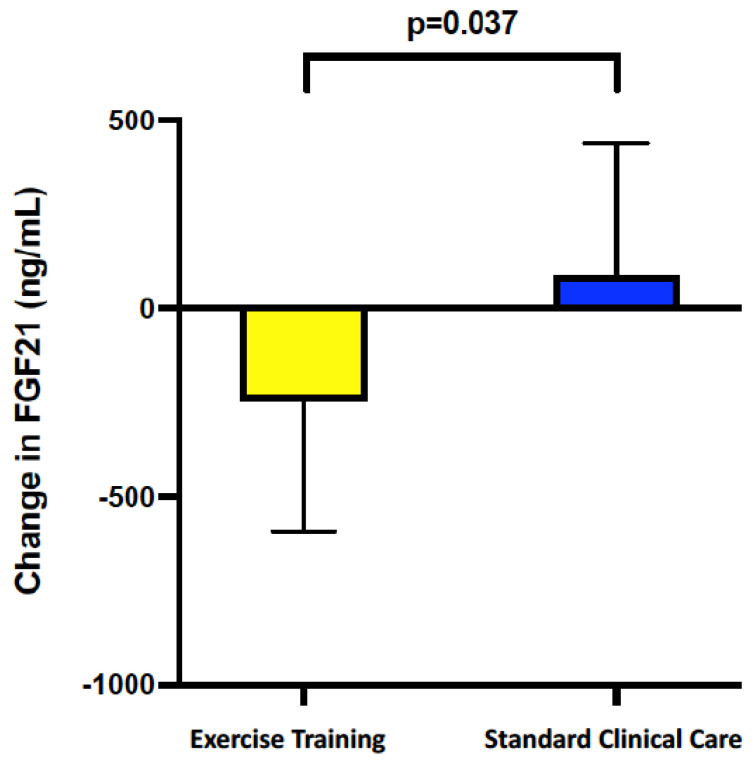
Change in serum FGF21 comparing exercise training to standard clinical care. Serum FGF21 was reduced by 22% (−243 ng/mL) following exercise training, whereas standard clinical care patients had an increase of 34% (+88 ng/mL).

**Figure 2:**
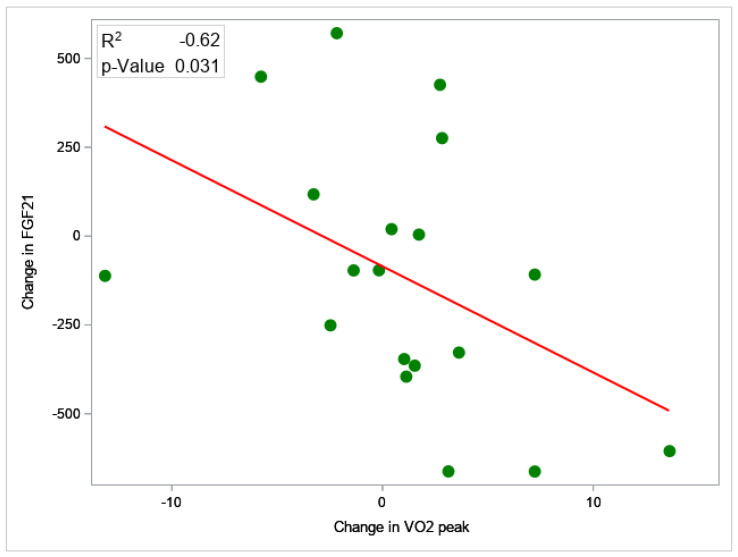
Correlation between change in serum FGF21 and VO_2_peak. Serum FGF21 reduction was significantly correlated with VO_2_peak gain (r = −0.62, 95% CI −0.88 to −0.05, *p* = 0.031). On multivariable analysis, VO_2_peak improvement remained independently associated with FGF21 decrease (β = −44.5, 95% CI −83.8 to −5.11, *p* = 0.031).

There was a significant improvement in serum FGF21 with exercise training compared with standard clinical care (*p* = 0.037). In patients who underwent exercise training, serum FGF21 was reduced by −22% (−243.4 ng/mL, 95% CI −441.4 to −45.5 ng/mL), whereas standard clinical care patients had a +34% increase (+88.4 ng/mL, 95% CI −90.0 to + 314.5 ng/mL, *p* = 0.037) (Figure 1). Serum FGF21 reduction was significantly correlated with VO_2_peak gain (r = −0.62, 95% CI −0.88 to −0.05, *p* = 0.031), liver volume reduction (r = 0.68, 95% CI 0.18 to 0.89, *p* = 0.016), and PAI-1 reduction (r = 0.62, 95% CI 0.04–0.87, *p* = 0.033) (Figure 2). On multivariable analysis, VO_2_peak improvement remained independently associated with FGF21 decrease (β = −44.5, 95% CI −83.8 to −5.11, *p* = 0.031). This means that for every one unit increase in VO_2_peak, serum FGF21 will reduce by 44.5 ng/mL.

### 3.3. Change in Non-Invasive Tests for NASH

Several significant changes were seen in serum and imaging biomarkers following exercise training (Table 2). MRI-PDFF was reduced by −5.0% (95% CI −8.2 to −1.8%) following exercise training, while patients in the standard of care arm experienced a +1.2% (95% CI −0.7 to +3.1%) gain in liver fat (*p* = 0.011). In all, 33% of exercise training patients met the minimal clinically important difference of at least 30% relative reduction in MRI-PDFF [28,29], which surrogates for improvement in histologic NASH activity and liver fibrosis, compared to 13% of standard of care patients (*p* = 0.008). In total, 58% of exercise training patients achieved at least a 17 IU/L reduction in alanine aminotransferase (ALT) [30], which also surrogates for liver fibrosis improvement, compared to 13% of standard clinical care patients (*p* < 0.001). All four of the patients who achieved at least 30% relative reduction in MRI-PDFF also had at least a 17 IU/L reduction in ALT.

Serum biomarkers were also improved, including a reduction in plasminogen activator one (PAI-1) of −45 ng/mL (95% CI −106 to +15 ng/mL) compared to a +70 ng/mL (95% CI +19 to +106 ng/mL) increase in the standard of care condition (*p* = 0.020) as well as a reduction in cytokeratin (CK)−18 of −59 IU/L (95% CI −86 to −30 IU/L) versus a +70 IU/L gain (95% CI −28 to +168 IU/L) with standard clinical care (*p* = 0.062). The FGF21/adiponectin ratio in the exercise group was reduced compared to the standard of care group (−0.07, 95% CI −0.13 to −0.01 vs. +0.02, 95% CI −0.07 to +0.11, respectively, *p* = 0.099). No statistically significant change was observed in serum biomarker adiponectin or clinical decision aids NAFLD Fibrosis Score or the Fibrosis-4 index.

### 3.4. Change in Non-Hepatic Clinical Outcomes

Cardiorespiratory fitness and glycemic control were significantly improved following exercise training where patients in the exercise arm experienced a +2.8 mL/kg/min gain (95% CI +0.1 to +5.5 mL/kg/min) in VO_2_peak compared to a −1.9 mL/kg/min loss (95% CI −5.4 to +1.7 mL/kg/min) with standard clinical care (*p* = 0.057) (Table 3). In all, 50% of patients achieved a clinically significant improvement in VO_2_peak of at least 10% following exercise training compared to 0% of patients in the standard of care arm.

Hemoglobin A1c was improved by −0.5% (95% CI −0.8 to −0.2%) with exercise training compared to a +0.4% (95% CI 0.0% to +0.8%) gain in the standard of care arm (*p* = 0.006), corresponding to similar changes in fasting serum glucose (−19.5 mg/dL, 95% CI −38.2 to −0.8 mg/dL exercise vs. +20.2 mg/dL, 95% CI −9.3 to +49.7 mg/dL standard clinical care, *p* = 0.030). While not statistically significant, insulin resistance as measured by homeostatic model assessment for insulin resistance (HOMA-IR) improved following exercise training (−5.5, 95% CI −13.3 to +2.3, *p* = 0.148). Modest weight change was observed following exercise training −2.1 kg (95% CI −4.4 to +0.2 kg), but this was not statistically significant (*p* = 0.705) nor clinically meaningful, as this was a <3% relative reduction.

## 4. Discussion

This post hoc analysis of the NASHFit trial found serum FGF21 to be markedly decreased following 20 weeks of moderate-intensity aerobic exercise training and without clinically significant body weight loss compared with standard clinical care. This finding supports a novel mechanism to explain the observed reduction in MRI-measured liver fat and improvement in serum biomarkers of liver fibrosis in patients with NASH who do undertake aerobic exercise. This is the first study to show that improvement in serum FGF21 is sustained with a long-term exercise training in patients exclusively with NASH, extending the findings of previous study by Taniguchi et al. of 27 elderly Japanese men without established NAFLD which found a short-term five-week aerobic exercise program to decrease serum FGF21 [21]. Taken together, regular aerobic exercise appears to improve the FGF21 resistant-state characteristic of NASH and lead to measurable clinical benefits at or above the thresholds of meaningful response.

FGF21 regulates energy metabolism and is largely expressed in the liver, although it can be found in other tissues, including adipose tissue [31]. In the liver, FGF21 stimulates fatty acid oxidation while simultaneously inhibiting de novo lipogenesis [32], an effect that may be mediated through changes in the AMP-activated protein kinase (AMPK) pathway [33,34]. It is plausible that exercise-induced reduction in serum FGF21 may feed back to the liver and potentially stimulate hepatic expression of FGF21, leading to these favorable changes in metabolism and liver fat. FGF21 is also closely related to insulin resistance. Whether the benefits of an exercise-induced reduction in serum FGF21 act directly on the liver or are instead mediated through improvement in insulin resistance, which we observed with measurable improvement in glycemic control and reduction in HOMA-IR, is not possible to answer through this study yet offers an intriguing avenue for future study and would require liver histology.

The results of a recent study [23] show that the ratio of FGF21/adiponectin is higher in subjects with NAFLD than in those without NAFLD, and that there was a positive relationship between change in the ratio and liver fat percentage in individuals enrolled in a clinical weight loss program. Based on these results, the authors propose that the FGF21/adiponectin ratio is a potential biomarker to monitor changes in liver fat content. Interestingly, in the present study, there was a trend towards a decrease in the FGF21/adiponectin ratio in the exercise group compared to the standard of care group, consistent with the observed reduction in MRI-measured liver fat. Future studies are warranted to assess the FGF21/adiponectin ratio as a biomarker for exercise-induced reductions in liver fat and to better understand if exercise training restores normal function in the FGF21–adiponectin axis.

The relationship between FGF21 and cardiorespiratory fitness is also of great interest given that VO_2_peak is associated with histologic NASH activity and liver fibrosis [35,36] and also overall mortality in the general population [37] as well as NAFLD [38]. Previous studies have not only demonstrated an association between cardiorespiratory fitness and serum FGF21 [39] but also that FGF21 is predictive of future adverse cardiovascular disease events [40]. Whether a reduction in serum FGF21 and, in effect, the prevention of FGF21 resistance in patients with NASH lowers the risk of future cardiovascular disease events remains unknown but of great significance given that cardiovascular disease is a leading cause of morbidity and mortality in patients with NAFLD and NASH [2]. The multivariable regression demonstrated that a 1 mL/kg/min improvement in VO_2_peak was associated with a 44.5 ng/dL decrease in serum FGF21. This magnitude of change in VO_2_peak is frequently observed with even modest amounts of aerobic exercise. Moderate-intensity aerobic exercise training studies of 4–16 weeks duration have shown an average improvement of 3.6 mL/kg/min in people with NAFLD [41]. While the minimally clinically important difference for change in FGF21 is unknown, this magnitude of change in VO_2_peak would lead to a ~30% improvement in serum FGF21 in the NASHFit cohort.

FGF21 is also implicated in the development of extrahepatic cancers found more commonly in patients with NAFLD, including breast, colorectal, esophageal, and pancreatic, as well as hepatocellular carcinoma [42]. Because extrahepatic and hepatic cancers are also leading causes of death in patients with NAFLD and NASH, the ability of exercise to improve FGF21 resistance offers promise as a mechanism of interest to explore as we continue to tease out the protective benefit of regular physical activity on oncologic risk [43,44].

This study has multiple strengths in that it uses paired samples from a highly rigorous clinical trial conducted in a population of patients with NASH who were well phenotyped and studied systematically. Possible limitations include the sample size (although this study is powered similar to previously published exercise trials in patients with NAFLD), the lack of liver histology, the inability of the study design to evaluate long-term clinical outcomes, and the fact that serum FGF21 was not possible to measure for each patient who completed the NASHFit trial.

## 5. Conclusions

Serum FGF21 is markedly decreased in response to exercise training, offering a novel mechanism to explain the observed reduction in liver fat, improvement in serum biomarkers of liver fibrosis, and gains in cardiorespiratory fitness in patients with NASH who do exercise. Future studies are required to determine if exercise training can directly impact patient outcomes by ameliorating the FGF21-resistant state that is characteristic of NASH.

## Figures and Tables

**Table 1:** Baseline comparisons between Exercise and Standard Clinical Care participants.

KERRYPNX	Standard Clinical Care (*n* = 8)	Exercise (*n* = 12)
Demographics		
Age, yrs	44.9 (36.8, 55.0)	53.9 (45.7, 63.6)
Female sex, *n* (%)	3 (38)	8 (67)
BMI, kg/m^2^	34.2 (29.9, 39.1)	32.8 (29.7, 36.2)
Body weight, kg	106.4 (89.1, 127.2)	95.5 (87.4, 104.3)
Metabolic risk		
Comorbidities, *n* (%)		
Diabetes	3 (38)	5 (42)
Hyperlipidemia	5 (63)	7 (58)
Hypertension	5 (63)	12 (100)
Hemoglobin A1c, %	6.1 (5.3, 7.0)	6.4 (5.6, 7.4)
Glucose (fasting), mg/dL	128.4 (97.1, 169.7)	136.3 (115.2, 161.3)
HOMA-IR	7.7 (4.0, 15.0)	13.3 (9.2, 19.2)
VO_2_peak, mL/kg/min	24.0 (19.8, 29.1)	19.3 (15.8, 23.6)
Body fat, %	39.1 (30.8, 49.7)	42.3 (37.7, 47.5)
NASH phenotyping		
Medications, *n* (%)		
Vitamin E	2 (25)	3 (25)
GLP-1 agonist	0 (0)	1 (8)
Non-invasive tests		
FIB-4	1.07 (0.47, 2.40)	1.15 (0.85, 1.57)
NFS, Median (IQR)	−1.75 (2.74)	−1.51 (1.25)
Serum biomarkers		
Adiponectin, ng/mL	3272 (2211, 4843)	3449 (2947, 4036)
CK-18, IU/L	85.7 (31.1, 236.4)	231.5 (76.3, 702.1)
FGF-21, ng/dL	372.4 (246.8, 561.8)	520.7 (346.1, 783.3)
PAI-1, ng/mL	206.0 (120.1, 353.5)	165.8 (124.0, 221.7)
Imaging biomarkers		
Liver fat (MRI-PDFF), %	19.0 (11.8, 30.6)	20.5 (17.4, 24.2)
NAS	5.0 (4.5, 5.4)	5.1 (4.5, 5.7)
Steatosis	2.3 (2.0, 2.7)	2.5 (2.0, 3.0)
Lobular inflammation, Median (IQR)	1.0 (1.0)	1.3 (0.7)
Hepatocyte ballooning	1.2 (0.9, 1.5)	1.2 (1.0, 1.5)
Fibrosis stage, *n* (%)		
0/1	4 (50)	7 (58)
2	3 (38)	2 (17)
3	0 (0)	3 (25)
4	1 (12)	0 (0)

BMI = body mass index; GLP = glucagon-like peptide; HOMA-IR = homeostatic model assessment for insulin resistance; MRI = magnetic resonance imaging; NAFLD = nonalcoholic fatty liver disease; NAS = NAFLD Activity Score; PDFF = proton density fat fraction; VO_2_ = oxygen consumption. Continuous variables reported as geometric mean (95%CI) unless indicated. No subjects were taking pioglitazone or obeticholic acid. All *p*-values were >0.05 for baseline characteristics between groups.

**Table 2:** Outcome measures: Non-invasive tests.

	Standard Clinical Care (*n* = 8)			Exercise (*n* = 12)			
	Baseline	Post	Within Group *p*-Value	Baseline	Post	Within Group *p*-Value	Between Group *p*-Value
Clinical Decision Aids							
NFS, Median (IQR)	−1.75 (2.74)	−1.42 (2.60)	0.653	−1.51 (1.25)	−1.69 (1.30)	0.679	0.431
FIB-4	1.07 (0.48, 2.40)	1.02 (0.47, 2.20)	0.671	1.15 (0.85, 1.57)	1.03 (0.76, 1.40)	0.077	0.777
Serum Biomarkers							
Adiponectin, ng/mL	3271 (2210, 4843)	3141 (2101, 4698)	0.468	3448 (2947, 4035)	3530 (3060, 4071)	0.652	0.440
CK18, IU/L	85.7 (31.1, 236.4)	85.8 (16.1, 458.1)	0.998	231.5 (76.3, 702.1)	177.0 (56.7, 552.6)	0.034	0.062
FGF21, ng/mL	372 (247, 562)	390 (190, 802)	0.670	521 (346, 783)	277 (153, 501)	0.044	0.037
FGF21/Adiponectin	0.11 (0.06, 0.19)	0.13 (0.07, 0.27)	0.602	0.15 (0.09, 0.24)	0.07 (0.04, 0.15)	0.049	0.099
PAI-1, ng/mL	206 (120, 353)	279 (187, 415)	0.061	166 (124, 221)	130 (101, 168)	0.160	0.020
≥17 IU/L reduction in ALT, *n* (%)		1 (13)			7 (58)		<0.001
Imaging biomarkers							
MRI-PDFF liver fat, %	19.0 (11.8, 30.6)	18.8 (10.2, 34.7)	0.934	19.0 (11.8, 30.6)	18.8 (10.2, 34.7)	0.018	0.011
≥30% relative reduction in MRI-PDFF, *n* (%)		1 (13)			4 (33)		0.008

ALT = alanine aminotransferase; CK = cytokeratin; FGF = fibroblast growth factor, FIB-4 = fibrosis-4 index; MRI = magnetic resonance imaging; NAFLD = nonalcoholic fatty liver disease; NFS = NAFLD Fibrosis Score; PAI = plasminogen activator inhibitor, PDFF = proton density fat fraction. Reported as geometric mean (95% CI) unless indicated.

**Table 3:** Outcome measures: Anthropometry and body composition, fitness, biochemistry and lipids.

	Standard Clinical Care (*n* = 8)			Exercise (*n* = 12)			
	Baseline	Post	Within Group *p*-Value	Baseline	Post	Within Group *p*-Value	Between Group *p*-Value
Anthropometry and Body composition							
BMI, kg/m^2^	34.2 (29.9, 39.1)	34.6 (30.3, 39.5)	0.173	32.8 (29.7, 36.2)	32.2 (29.2, 35.4)	0.267	0.155
Body weight, kg	106.6 (89.1, 127.2)	107.9 (90.1, 129.3)	0.069	95.5 (87.4, 104.3)	93.4 (85.8, 101.8)	0.099	0.030
Waist circumference, in	45.5 (41.6, 49.9)	46.1 (42.3, 49.9)	0.385	43.8 (41.6, 46.1)	43.1 (40.9, 45.4)	0.230	0.135
Hip circumference, in	45.3 (40.7, 50.5)	46.3 (41.6, 51.6)	0.001	44.9 (42.4, 47.5)	43.6 (40.5, 47.5)	0.243	0.026
VAT, lbs.	6.5 (4.7, 8.9)	6.8 (4.7, 9.7)	0.795	5.5 (4.7, 6.4)	5.1 (4.3, 5.9)	0.016	0.122
Fat free (muscle) mass, lbs.	117.2 (88.8, 154.7)	122.1 (88.7, 154.7)	0.294	106.6 (87.1, 130.4)	106.5 (87.1, 130.3)	0.913	0.611
Body fat, %	39.1 (30.8, 49.7)	37.1 (28.7, 48.1)	0.259	42.3 (37.7, 47.5)	41.4 (36.5, 46.8)	0.040	0.254
Liver volume, cc	2480 (2028, 3031)	2543 (2116, 3055)	0.289	2305 (2022, 2627)	2118 (1861, 2410)	0.020	0.024
Cardiorespiratory fitness							
Resting VO_2_, L/min	3.4 (2.7, 4.2)	3.5 (3.0, 4.0)	0.432	3.5 (2.7, 4.5)	4.0 (3.3, 4.8)	0.378	0.573
VO_2_peak, mL/kg/min	24.0 (19.8, 29.1)	22.5 (20.0, 25.4)	0.381	19.3 (15.8, 23.6)	22.6 (20.0, 25.5)	0.076	0.057
Biochemistry							
ALT, IU/L	51.6 (40.0, 66.6)	50.7 (20.0, 25.4)	0.318	56.8 (46.8, 68.9)	43.8 (35.7, 53.7)	0.009	0.259
AST, IU/L	39.4 (27.2, 57.1)	33.4 (25.0, 44.7)	0.388	38.9 (32.1, 47.1)	30.6 (25.4, 36.8)	0.004	0.859
Glucose (fasting), mg/dL	128.3 (97.1, 169.7)	139.1 (93.0, 207.9)	0.386	136.3 (115.2, 161.3)	117.4 (100.2, 137.7)	0.074	0.030
Hemoglobin A1c, %	6.1 (5.3, 7.7)	6.4 (5.4, 7.7)	0.056	6.4 (5.6, 7.4)	6.1 (5.5,6.6)	0.028	0.006
HOMA-IR	7.7 (4.0, 15.0)	7.7 (3.2, 18.7)	0.880	13.3 (9.2, 19.2)	7.9 (4.8, 13.2)	0.080	0.168
Insulin, IU/mL	24.4 (15.4, 38.8)	22.5 (10.9, 46.2)	0.447	39.5 (28.0, 55.7)	27.4 (15.9, 47.0)	0.152	0.351
Lipids							
Total cholesterol, mg/dL	199.5 (169.5, 234.7)	182.5 (160.7, 207.3)	0.358	183.1 (157.8, 212.4)	183.4 (160.2, 210.0)	0.969	0.410
LDL, mg/dL	114.9 (82.2, 160.5)	104.3 (72.9, 149.4)	0.510	102.8 (82.4, 128.4)	101.9 (83.0, 125.0)	0.906	0.583
HDL, mg/dL	41.5 (32.3, 53.4)	37.9 (28.0, 51.4)	0.352	41.7 (36.1, 48.1)	43.7 (39.2, 48.6)	0.221	0.152
Triglyceride, mg/dL	207.5 (144.1, 298.7)	210.2 (134.1, 329.5)	0.974	172.3 (137.3, 216.4)	168.8 (130.4, 218.5)	0.781	0.309

ALT = alanine aminotransferase; AST = aspartate aminotransferase, BMI = body mass index; HDL = high-density lipoprotein; HOMA-IR = homeostatic model assessment of insulin resistance; LDL = low-density lipoprotein; VAT = visceral adipose tissue; VO_2_ = oxygen uptake. Reported as geometric mean (95% CI) unless indicated.

## Data Availability

The data presented in this study are available on request from the corresponding author.

## Abbreviations

ALT = alanine aminotransferase; AMPK = adenosine monophosphate activated protein kinase; BMI = body mass index; CK = cytokeratin; ELISA = enzyme-linked immunoassay; FGF = fibroblast growth factor; MRI = magnetic resonance imaging; NAFLD = nonalcoholic fatty liver disease; NASH = nonalcoholic steatohepatitis; PAI = plasminogen activator inhibitor; PDFF = proton density fat fraction; SD = standard deviation; VO_2_ = oxygen uptake.
